# Intelligence

**Published:** 1808-10

**Authors:** 


### INTELLIGENCE.

Professor Davy has read a paper before the Royal Society, containing an account of his various new and important electrical experiments on the decomposition of the earths (already detailed in this Journal,) by which this distinguished philosopher has shewn that they are all metallic oxides, and has thus verified by experiment what had been long suspected from analogy. These important discoveries complete the history of alkaline and earthy bodies, and form an era in chemical philosophy. They likewise must lead to great improvements in mineralogy and geology, the last of which sciences has hitherto wanted elements. In the same communication in which these facts are announced, a most important experiment of two Swedish chemists, Messrs. Benzelius and Pontin, is mentioned. These gentlemen have combined the basis of volatile alkali with quicksilver, and in this way have formed an amalgam. Here is something metallic apparently composed of two gases, a circumstance in which the dreams of the alchemists seem to be realized.

The Rev. Mr. Leg, of Ashprington, Devon, has invented a contrivance for discharging the superfluous water from ponds, tanks, and reservoirs, in times of floods. It consists of a beam of wood, suspended by an iron pin, over the head of the water, so as to form a kind of lever or balance, having one end affixed to a chain, which raises a plug, to let out the superfluous water, and having suspended at the other a box or bucket, made rather leaky, into which the water is conveyed by a pipe, whenever it rises to a certain tain level. As long as the bucket continues filled with water, the weight will raise the plug, and when the water no longer keeps the bucket full, the plug will return to its place, by the lever recovering its level position.

Dr. Parry, of Bath, has recently investigated the causes of the decay of wood, and the means of preventing it. For this purpose, he recommends the application of a preparation of the rezinous kind mixed with a certain portion of bees-wax. The proportion of the ingredients, and the mode of mixing them, are as follow.—Take twelve ounces of rosin, and eight ounces of roll brimstone, each coarsely powdered, and three gallons of train-oil; heat them slowly, gradually adding four ounces of bees-wax, cut in small bits. Frequently stir the liquor, which, as soon as the solid ingredients are dissolved, will be fit for use. It is recommended to dress every part of the wood work with this composition, twice over before the parts are put together, and once afterwards; and a higher state of preservation is promised from its use than has yet been attained. It should be observed, that in preserving this varnish, it is adviseable, in order to prevent accidents, to use an earthen vessel, and to make the fire in the open air.

The following simple method of preventing the destruction of flax by the caterpillar, has been practised with success in Holland. It consists in making persons walk twice a day along the furrows, with a rope fastened to two poles, so as to cause the rope to drag over the plants, by which the insects are swept off them. This operation repeated for four succeeding days preserves the flax; though, in some instances, in three days it has been found to produce the desired effect.

An interesting analysis of coffee has recently been made by M. Cadet, apothecary in ordinary to the French imperial household, from which it appears that the berries contain mucilage in abundance, much gallic acid, a resin, a concrete essential oil, some albumen, and a volatile aromatic principle. To these may be added such as are found in most vegetables, viz. lime, pot-ash, charcoal, iron, &c. Roasting developes the soluble principles; but it ought to be moderated, if it be wished to preserve the aroma, and not decompose the acid, the gum and the resin. Mocha coffee is of all kinds the most aromatic and resinous. M. Cadet advises that coffee be neither roasted nor infused till the day it is intended to be drunk.

The greater part of the celebrated vineyards of Tokay have this year been entirely destroyed by a tremendous storm, accompanied with hail and the bursting of a cloud. The hailstones were as large as walnuts, and the bursting of the cloud was so violent, that stones of great weight were thrown from the vineyards into the village. By this accident seven men and one boy lost their lives, and a great number of cattle perished. The injury sustained by the vineyards is incalculable. The whole village was so entirely filled filled with stones, that the labour of hundreds of people was required to clear them away.

The Legislature of Maryland have passed an act for founding a medical college in the city or precincts of Baltimore, for the instruction of students in the different branches of medicine. This institution is established upon a liberal plan, and incorporated in perpetuity. It consists of a board, called the Regents of the College of Medicine of Maryland, formed from the existing board of medical examiners for the commonwealth, and the president and professors appointed by the act. It may hold property to a value not exceeding thirty thousand dollars, exclusive of a lot of buildings. The regents may appoint professors and lecturers, who shall form one learned body, under the name of the Medical Faculty, with power to chuse their dean, and to do what is necessary for conveying instruction, and supporting discipline. The regents must meet at least once a year. The faculty shall hold at least one term annually, to begin on the first Monday in November, and continue not less than four, nor more than six months. At convenient times commencements may be held, and degrees in surgery and medicine may be granted, after due examination and other proofs of sufficiency. Each student must have attended each course of lectures at least once, and frequented the classes of the college for two terms: and he must also have been privately and publicly examined, and have printed and defended a thesis, before he can be admitted to the honours of the college.

It has been suggested that the sunflower might be successfully cultivated for the purpose of supplying our clothiers with oil. As much of the oil imported from the Levant, under the name of Florence oil, when it becomes rancid, is sold to the clothiers for the purpose of softening their wool, when preparing for the loom, it is conceived that the oil extracted from the seed of the sun-flower might be advantageously employed in the same way.

---

St. George's Hospital, and George-Street, Hanover Square. — On *Thursday, October* 6, Dr. George Pearson, F. R. S. Senior Physician to St. George's Hospital, of the College of Physicians, &c. will recommence a Course of Lectures on Physic and Chemistry, at the Laboratory, George-street, Hanover-square, at the usual morning hours; viz. the Therapeutics, at a quarter before eight: the Practice of Physic at half after eight; and the Chemistry at a quarter after nine. Clinical Lectures are given, as usual, on the patients of St. George's Hospital, every Saturday morning, at nine o'clock. — Proposals to be had in George-street, or at St. George's Hospital.

Mr. Chevalier will commence his next Course of Lectures on the Principles and Operations of Surgery, on Wednesday, the 5th of October, at eight o'clock in the evening, at his house in South Audley-street, Grosvenor-square.

Dr.

Dr. CLUTTERBUCK will begin his Lectures on the Theory and Practice of Physic and the Materia Medica, on Tuesday, Oct. 4, at ten o'clock in the forenoon, at the General Dispensary, Aldersgate-street.

Mr. CARLISLE, F. R. S. and Surgeon to the Westminster Hospital, will resume his Course of Lectures on the Art and Practice of Surgery, in the beginning of October, at his house in Soho-square; other particulars may be known by enquiring at Mr. C's house.

---

A MOST useful and elegant Anatomical Work, in folio, will be published in a few days, entitled Anatomico-Chirurgical Views of the Nose, Mouth, Larynx, and Fauces, with appropriate Explanations and References to the Parts. By Mr. J. J. Watt, Surgeon; designed by the Author, to illustrate the Anatomy of these Organs, as they appear in different sections of the head, and performed with the strictest attention to anatomical accuracy. The engravings will be four in number, containing six views of the parts of their natural size, and accompanied with the same number of outline figures of reference; with an additional anatomical description of these organs. By Mr. W. LAWRENCE, Demonstrator of Anatomy, at St. Bartholomew's Hospital.

Dr. ANDREW GRANT, who has recently returned from South America, has in the press a History of Brazil, which will contain a geographical and historical account of that important colony, with a description of the manners, customs, religion, &c. of the natives; interspersed with remarks on the nature of its soil, climate, productions, and foreign and internal commerce; to which will be subjoined, observations on the most prevalent diseases incident to the climate, with hints to new settlers on the most efficacious modes of prevention. It will form one volume, octavo.

Dr. SMITH will shortly publish a work in one volume octavo, under the title of Botanical Illustrations, intended as a continuation of his Introduction to Botany.

